# 575. Optimization of a Nitrocefin-Based Rapid Test for the Detection of the Cefazolin Inoculum Effect (CzIE) in Methicillin-Susceptible *Staphylococcus aureu*s

**DOI:** 10.1093/ofid/ofad500.644

**Published:** 2023-11-27

**Authors:** Sara I Gomez Villegas, Diana Panesso, Kavindra Singh, Sandra Rincon, Lina Paola Carvajal, Karen Jacques-Palaz, Samie Rizvi, Carey-Ann Burnham, April Abbott, Jennifer Dien Bard, Lars Westblade, Stephanie A Fritz, Barbara Zimmer, Tanis C Dingle, Susan Butler-Wu, William R Miller, Jinnethe Reyes, Cesar A Arias

**Affiliations:** Brigham and Women's Hospital - Harvard Medical School, Boston, Massachusetts; Houston Methodist Research Institute, Houston, Texas; Houston methodist research institute, Houston, Texas; Universidad El Bosque, Bogota, Distrito Capital de Bogota, Colombia; Universidad El Bosque, Bogota, Distrito Capital de Bogota, Colombia; Houston Methodist Research Institute, Houston, Texas; Houston Methodist Research Institute, Houston, Texas; Washington University, St. Louis, MO; Deaconess Health system, Evansville, Indiana; Children’s Hospital Los Angeles, Los Angeles, California; Weill Cornell Medicine, New York, NY; Washington University School of Medicine, St. Louis, MO; Beckman Coulter, West Sacramento, CA; University of Alberta, Alberta Precision Labs, Edmonton, Alberta, Canada; Keck School of Medicine of USC, CA; Houston Methodist Research Institute, Houston, Texas; Universidad El Bosque, Bogota, Distrito Capital de Bogota, Colombia; Houston Methodist and Weill Cornell Medical College, Houston, TX

## Abstract

**Background:**

The cefazolin (Cz) inoculum effect, defined as a Cz MIC ≥16 µg/mL at 10^7^ CFU/mL in MSSA isolates, has been associated with poor outcomes in adult patients with MSSA bacteremia and pediatric patients with osteomyelitis. Broth microdilution at high inoculum is the gold standard for the detection of the CzIE, but this method is time and labor-intensive. We developed a nitrocefin-based rapid test that can identify the CzIE among MSSA(Figure 1). However, the original protocol for this rapid test includes the use of ampicillin in solution, which is difficult to prepare and unstable once reconstituted. In this study, we aimed to show that simple elution from ampicillin disks available in the typical clinical microbiology lab performs as well as an ampicillin powder-based assay.

**Figure 1.**
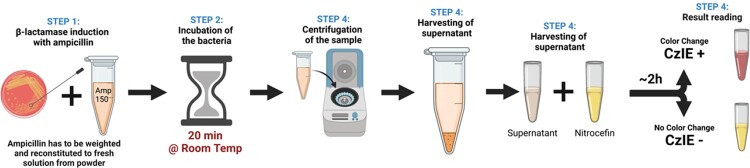
Original Protocol Nitrocefin Rapid Test

Original Protocol for the performance of the Nitrocefin-based rapid test as published by Rincon et at (Rincon et al, JCM, 2021)

**Methods:**

We included 107 unique MSSA bloodstream isolates which had been previously characterized (Dingle et al., *JCM*, 2022). The CzIE was determined using gold standard broth microdilution at high inoculum (10^7^ CFU/mL). The published protocol of the nitrocefin test (Rincon et al., *JCM*, 2021) was modified as follows (Figure 2): instead of adding ampicillin from powder to BHI broth (150 ug/mL), ampicillin disks (10 ug) of 2 different brands (Oxoid and BD) were added to 1 mL of BHI broth of 2 different brands (Oxoid, BD) and incubated for 2 hours at room temperature with frequent shaking (Figure 3). The remaining steps of the protocol were not modified. The original protocol was used as control. Performance metrics were calculated for the complete data set.

**Figure 2.**
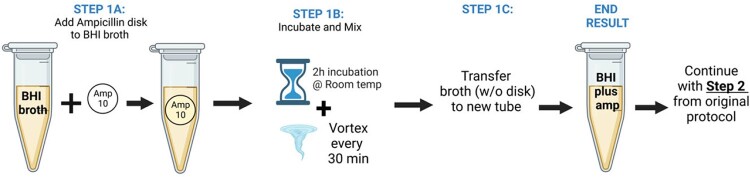
Modification to Nitrocefin-based rapid test protocol

Modification of the Step 1 from the original protocol to include ampicillin disks instead of ampicillin from powder.

**Figure 3.**
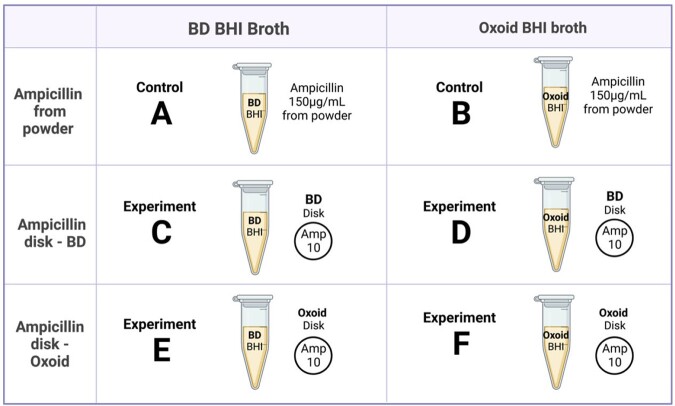
Experiments and Controls.

2 different brands of BHI broth and 2 different brands of ampicillin disks(10 ug) were compared to the use of ampicillin from powder to a final concentration of 150 ug/mL

**Results:**

51.4%(55/107) of the isolates were CzIE+ by reference broth microdilution at high inoculum. When using Oxoid BHI broth, the sensitivity and specificity of the test were 96.3% and 90.3% regardless of the ampicillin form (disk vs powder) or disk brand (Table 1). When using BD BHI broth, the sensitivity and specificity of the test were 69.1% and 94.2%, regardless of the ampicillin form or disk brand.

**Table 1.**
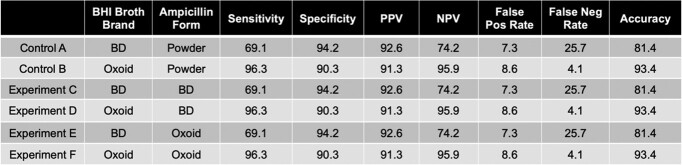
Results Performance metrics of the Nitrocefin-based rapid test with the original protocol (Control A and Control B) and the modified protocol using ampicillin disks from 2 different brand (BD: Experiments C and D; Oxoid: Experiments E and F) as well as 2 different brands of BHI broth (BD: Control A, experiments C and E; Oxoid: Control B, experiments D and F)

**Conclusion:**

The modification of the nitrocefin-based rapid to use ampicillin disks instead of ampicillin powder did not impact the accuracy of the tests as it was able to correctly detect the CzIE in isolates, regardless of the brand of ampicillin disk. Implementation of this test may contribute to therapeutic decisions in deep-seated MSSA infections.

**Disclosures:**

**Jennifer Dien Bard, PhD**, Abbott Molecular: Grant/Research Support|BioMerieux: Advisor/Consultant|BioMerieux: Grant/Research Support|BioMerieux: Honoraria|Genetic Signature: Advisor/Consultant|Genetic Signature: Grant/Research Support|Luminex: Grant/Research Support|Salve: Stocks/Bonds|Thermo Fisher: Honoraria **Lars Westblade, PhD**, Accelerate Diagnostics, Inc.: Grant/Research Support|bioMerieux, Inc.: Grant/Research Support|Hardy Diagnostics: Grant/Research Support|Roche Molecular Systems, Inc.: Advisor/Consultant|Roche Molecular Systems, Inc.: Grant/Research Support|Selux Diagnostics, Inc.: Grant/Research Support|Shionogi, Inc.: Advisor/Consultant|Talis Biomedical: Advisor/Consultant **William R. Miller, M.D.**, Merck: Grant/Research Support|UpToDate: Honoraria

